# The association between TP53 rs1625895 polymorphism and the risk of sarcopenic obesity in Iranian older adults: a case-control study

**DOI:** 10.1186/s12891-021-04314-5

**Published:** 2021-05-13

**Authors:** Nima Montazeri-Najababady, Mohammad Hossein Dabbaghmanesh, Nasrin Nasimi, Zahra Sohrabi, Nazanin Chatrabnous

**Affiliations:** 1 Endocrinology and Metabolism Research Center, Shiraz University of Medical Sciences, Shiraz, Iran; 2 Nutrition Research Center, Shiraz University of Medical Sciences, Shiraz, Iran

**Keywords:** Sarcopenia, Obesity, p53, Polymorphism, Body mass index

## Abstract

**Background:**

Aging and obesity are the two major global health concerns. Sarcopenia, an age-linked disease, wherein a progressive loss of muscle volume, muscle strength, and physical activity occurs. In this study we evaluated the association of TP53 rs1625895 polymorphism with the susceptibility to sarcopenic obesity in Iranian old-age subjects.

**Methods:**

Total of 176 old individuals (45 sarcopenic and 131 healthy) were recruited in this research and genotyped by PCR–RFLP. BMI, Skeletal Muscle Mass Index, body composition, Handgrip Strength, Gait Speed (GS), and biochemical parameters were measured. Chi-square test was done for genotypes and alleles frequency. Linear regression was applied to find the correlation between TP53 rs1625895 polymorphism, and biochemical and anthropometric parameters. The correlation between TP53 rs1625895 and the risk of sarcopenia and sarcopenic obesity was investigated by logistic regression.

**Results:**

G allele was significantly higher in sarcopenic obesity group [*P* = 0.037, OR (CI 95%) = 1.9 (1.03–3.5)] compared to A allele. BMI (*P* = 0.049) and LDL (P = 0.04) were significantly differed between genotypes when GG was compared to AA/AG genotype. The results revealed when GG genotype compared to AA/AG genotype in adjusted model for age, the risk of sarcopenic obesity [*P* value = 0.011, OR (CI 95%); 2.72 (1.25–5.91)] increased. Similarly, GG/AG genotype increased the risk of sarcopenic obesity [*P* value = 0.028, OR (CI 95%); 2.43 (1.10–5.36)] in adjusted model for age compared to AA genotype.

**Conclusions:**

We suggested that TP53 rs1625895 polymorphism may increase the risk of sarcopenic obesity in Iranian population.

## Background

Aging and obesity are the two major global health concerns. Sarcopenia, an age-linked disease, wherein a progressive loss of muscle volume, muscle strength, and physical activity occurs [1]. Sarcopenia may also contributes to functional disability, fall incidence, hospitalization, stroke [2], decreased life quality [3], and augmented risk of morbidity and death [4] in elderly. Sarcopenia may develop in 15% of the individuals aged over 65 years and approximately 50% of subjects aged over 80 years [5]. According to the results of our previous study, sarcopenia was a prevalent condition and its prevalence was 20.8% among Iranian community-dwelling older adults [6].

The concurrence muscle weakness and accumulation of body fat mass is defined as sarcopenic obesity. Compared to sarcopenia or obesity alone, sarcopenic obesity results in a 2–3 times greater risk of disability, cardiovascular diseases, cognitive impairment, and morbidity and mortality of metabolic diseases [7]. The incidence rate of this complex disorder is approximately 5–10% of the elderly population which is more common in those aged over 80 years [8].

It is well known that genetic background, ecological factors and/or, interaction between gene and environment may be involved in developing variation in muscle phenotypes between individuals [9]. The epidemiological studies established that that genetic factors are responsible for 46–76% and 32–67% of fat-free mass [FFM] and muscle strength inconsistency, respectively [10]. So that, identification of novel candidate genes influencing skeletal muscle phenotypes is vital in designing an impressive treatment strategy to control age-associated variation in muscle arrangement and activity.

The TP53 gene is located on human chromosome 17 with the length of 16–20 kb which involves of 11 exons and 10 introns [11]. Recently, a considerable attention emphasis is on novel role (TP53) in developing cancer, in pathways related to metabolic traits such as obesity, insulin sensivity, inflammation of adipose tissue, and homeostasis of skeletal muscle [12, 13]. It was proposed that acute and quick degeneration of skeletal muscle, which represents for cachexia and of sarcopenia may be partly attributed to TP53 [14]. In addition, gene expression and structural analysis suggested that TP53 may be contributed to aging muscle and sarcopenia [15].

The intracellular signaling pathways such as programmed cell death [apoptosis]; increased protein deprivation through autophagy, calcium-dependent proteases (calpains and caspases), and proteasome system; decreased satellite cell activation accountable for muscle regeneration are closely related to the activity of p53 gene [16]. Based on the report of international agency for research on cancer [IARC; http://www.iarc.fr/index.php], the most common confirmed polymorphisms of TP53 are positioned at introns and a few ones in exons. rs1625895 is an intronic polymorphisms placed at intron 6 of TP53 transcript variant 1 composed of the allele A and allele G [17]. Evidence showed the association of rs1625895 with glioma and meningioma and also lung cancer [18, 19]. Also, a genetic study in the USA identified that TP53 rs1625895 polymorphism is associated with declined risk of aggressive breast cancer among women aged less than 50 years [20]. Furthermore, a Meta-analysis showed that TP53 rs1625895 variation is linked to ethnicity [21].

According to the proven importance of sarcopenia and sarcopenic obesity, the putative role of TP53 in regulation of muscle homeostasis, and the prevalence of sarcopenia among Iranian community-dwelling older adults reported in our previous study, for the first time we explored the genotype and allelic frequency of the TP53 rs1625895 [IVS6 + 62A > G] to outline if this polymorphism is associated with the susceptibility to sarcopenia and sarcopenic obesity in Iranian older adults’ population.

## Methods

The current study was approved by the Ethics local committee of Shiraz University of Medical Sciences (Shiraz, Iran) and have therefore been executed in accordance with the ethical standards Declaration of Helsinki (as revised in Brazil 2013). The present study was a subgroup of the cross-sectional, population-based study (from August 2017 to February 2018) used for evaluating the prevalence of sarcopenia and its determinants among Iranian old subjects which described previously [6]. Briefly, 501 older adults were chosen by clustered, stratified, multistage sampling based on geographical locations. The inclusion criteria of the study were being 65 years and older, independently living, physically active, and with no history of severe cardiac, pulmonary, or musculoskeletal diseases, severe nervous disorders, Parkinson, stroke, malignancies, or any other acute organs failure. In the current study, 176 individuals sarcopenia patients and 131 control) were randomly selected for genotyping. Our study was agreed by the ethics committee of the Shiraz University of Medical Sciences. The written informed consent for use of samples was obtained from all participants.

### Baseline characteristics

Body weight, height, waist, and calf circumference were defined by regular procedures. Age was obtained from questionnaire form interviewed. Body Mass Index (BMI) was measured by this formula: BMI (kg/m^2^) = weight (kg)/ [height (m)] ^2^ (Table 1).

**Table 1:** Comparison of the sarcopenic, sarcopenic obesity and healthy participants regarding baseline characteristics

Variables	Total (*n* = 206)	Sarcopenic (*n* = 45)	SO (*n* = 33)	Healthy (*n* = 131)	*P*-value^*^	*P*-value^**^
Age, years	70.45 ± 4.83	73.27 ± 5.94	70.0 ± 4.47	69.66 ± 4.16	**< 0.0001**	0.666
WC, cm	93.55 ± 9.05	87.28 ± 8.58	102.31 ± 7.46	93.60 ± 7.7	**< 0.0001**	**< 0.0001**
CC, cm	35.04 ± 3.27	31.93 ± 2.45	37.66 ± 3.10	35.46 ± 2.7	**< 0.0001**	**< 0.0001**
BMI, kg/m^2^	27.01 ± 4.45	23.47 ± 3.17	33.48 ± 3.16	26.7 ± 3.3	**< 0.0001**	**< 0.0001**
ALM, kg	19.03 ± 4.76	16.32 ± 3.28	15.62 ± 2.54	20.7 ± 4.84	**< 0.0001**	**< 0.0001**
FFM, kg	45.31 ± 8.28	40.31 ± 5.95	38.73 ± 4.45	48.4 ± 8.32	**< 0.0001**	**< 0.0001**
FM, kg	24.26 ± 8.22	19.39 ± 6.19	36.64 ± 5.39	23.1 ± 6.3	**< 0.0001**	**< 0.0001**
FM, %	34.63 ± 9.18	32.07 ± 7.71	48.51 ± 2.68	32.3 ± 7.7	**0.842**	**< 0.0001**
SMI, kg/m^2^	7.24 ± 1.12	6.33 ± 0.72	6.92 ± 0.80	7.6 ± 1.1	**< 0.0001**	**0.002**
HGS, kg	43.45 ±	33.87 ± 12.61	30.78 ± 9.24	49.5 ± 18.2	**< 0.0001**	**< 0.0001**
GS, m/s	17.98	0.70 ± 0.14	0.75 ± 0.18	0.93 ± 0.75	**0.046**	0.164
FM/FFM	0.86 ± 0.61	0.49 ± 0.18	0.94 ± 0.10	0.49 ± 0.16	0.989	**< 0.0001**
*Albumin, g/dl	4 (0.3)	3.9 (0.4)	3.9 (0.4)	4 (0.3)	**0.005**	**0.009**
*Creatinine, mg/dl	0.9 (0.2)	0.9 (0.2)	0.8 (18)	0.9 (0.15)	0.624	**0.008**
*BUN, mg/dl	14 (5)	15 (4)	13 (4)	14 (5)	0.904	0.138
*FBS, mg/dl	103.5	101 (20)	100.5 (18)	105 (20)	0.622	0.067
*Cholesterol, mg/dl	180 (57)	184 (66)	186.5 (71.5)	180 (56)	0.507	0.463
*LDL-C, g/dl	98.5 (52)	102 (63)	94 (55)	97 (49)	0.650	0.982
*HDL-C, mg/dl	50 (14)	49 (6)	59 (14.75)	50 (7)	0.878	**0.002**
*Triglycerides, mg/dl	125 (80.5)	107 (56)	120 (80)	132 (79)	0.110	0.205

Abbreviations: *SO* sarcopenic obesity; *WC* waist circumference; *CC* calf circumference; *BMI* body mass index; *FFM* fat-free mass; *FM* fat mass; *ALM* appendicular lean muscle mass; *SMI* skeletal muscle mass; *HGS* hand grip strength; *GS* gait speed

^*^ Comparison between sarcopenic and healthy participants

^**^ Comparison between sarcopenic obesity and healthy obesity participants

Independent t-test or Mann-Whitney U test was used. *P*-value<.0.05 are considered statistically significant

### Diagnostic measures for sarcopenia

The suggested diagnostic algorithm by Asian Working Group on Sarcopenia (AWGS) was applied to state sarcopenia among participants. According to this guideline, individuals with sarcopenia had low skeletal muscle mass and muscle function (low muscle strength or/and low physical performance). Skeletal muscle mass was assessed using a segmental multi-frequency Bioelectrical Impedance Analysis (BIA) In Body S10 analyzer (BioSpace Co., Ltd., South Korea) which measured segmental lean body mass, Fat-Free Mass (FFM), and body fat mass. Skeletal Muscle mass Index (SMI) was defined as a sum of arms and legs muscle mass (called Appendicular Lean Mass (ALM)) divided by the height squared (m^2^) and values less than 7.0 kg/m^2^ for male and 5.7 kg/m^2^ for female were reflected as low muscle mass [22].

Muscle function was appraised using Handgrip Strength (HGS) and usual Gait Speed (GS). HGS was measured using a hydraulic hand dynamometer (model MSD, Sihan, Korea) in sitting position. The mean of three measurements for both hands was calculated and HGS less than 26 kg for male and 18 kg for female were considered as a low muscle strength. GS was also assessed by a 4 m of independent walking and GS less than 0.8 m/s was considered as a low physical performance [22].

### Diagnostic measures for Sarcopenic obesity

The different diagnostic methods for determining obesity and sarcopenia have led to not having a comprehensive definition of sarcopenic obesity. However, among the traditional anthropometric measures to diagnose sarcopenic obesity, a higher fat mass to fat-free mass (FM/FFM) ratio is considered as a valuable diagnostic criterion when BIA is used for measurement. Accordingly, FM/FFM ratio more than 0.80 was considered as a sarcopenic obesity [23].

### Biochemical measurements

Blood samples were gathered after a 12 h fasting in hormone laboratory of the endocrinology and metabolism research center at Nemazee Hospital (an educational hospital affiliated to Shiraz University of Medical Sciences). Serum samples were collected two Eppendorf tubes, and stored at − 70 °C. Biochemical assessments were described previously [6]. Briefly, serum albumin, BUN, creatinine, Fasting Blood Sugar (FBS), and lipid profile (Triglyceride (TG), Low-Density Lipoprotein (LDL), High-Density Lipoprotein (HDL), and total cholesterol), were assessed by calorimetric assays using Biosystem SA auto-chemistry analyzer (DIRUI CS-T240, Spain).

### Snp selection and genotyping

TP53 rs1625895 (IVS6 + 62A > G) SNP selection was done based on the following criteria: 1. According to the minor allele frequency (MAF) of this SNP reported by TOPMED, PAGE_STUDY and 1000 genome, this is a common SNP (MAF > 0.1) in TP53 gene [24]. 2. A Previous study has shown that this is an intronic functional polymorphism of TP53 which can affect TP53 protein expression [25]. 3. A gene expression and structural analysis identified p53 as candidate gene that could be contributed in aging muscle and sarcopenia [15]. 4. Until now, no study investigated the association of this SNP with the risk of sarcopenia and sarcopenia obesity.

Blood samples for genotyping were collected after 10–12 h overnight fasting in tubes containing EDTA as an anticoagulant and kept at − 70 ∘C until extraction. Genomic DNA was mined from the whole blood by Cinnagen Kit DNP™ protocol (DNG plus DNA Extraction Kit, Cinnagen Company, Tehran, Iran). Polymerase chain reaction restriction–fragment length polymorphism (PCR–RFLP) was applied to explore to genetic variation of TP53 rs1625895. The primers nucleotide sequences were as follow:

Forward primer 5′- TCTGGTAAGGACAAGGGTTGG-3′,

Reverse primer 5′- GGAAGGGACAGAAGATGACAG-3′.

PCR settings for amplification of TP53 rs1625895 were 94 °C for 5 min (pre-denaturation), 35 cycles of 95 °C for 30 (denaturation), 59 °C for 30s (annealing), and 72 °C for 30s (extension), and completed with a 5-min final extension at 72 °C.

### Statistical analysis

Statistical results were achieved by IBM SPSS Statistics, version 22.0. Characteristics of participants were expressed as mean ± SD for continuous variables and percentage for categorical ones. The normal distribution of the data was assessed using Kolmogorov-Smirnov test. Levene’s test was conducted to assess the homogeneity of variance. Independent t-test or Mann-Whitney U test was used to compare the anthropometric and biochemical variables differences between the sarcopenic, sarcopenic obesity and healthy participants. Chi-square test or Kruskal-Wallis test was employed to compare the categorical variables. The frequency distribution of genotypes and alleles of TP53 rs1625895 in the sarcopenic, sarcopenic obesity, and healthy participants was evaluated for Hardy-Weinberg equilibrium (HWE) using chi-square tests. Linear regression analysis (adjusted for age) was directed to discover the association between TP53 rs1625895 polymorphisms, and anthropometric, and biochemical parameters. Binary logistic regression was also performed to determine the association between TP53 rs1625895 genotypes (AA/AG vs GG, and AG/GG vs AA) and the risk of sarcopenic obesity in unadjusted (model 1) and adjusted for age (model 2) models. Odds ratios (OR) and confidence interval (CI) 95% for the risk genotype for sarcopenic obesity were estimated. *P*-Values less than 0.05 were reported as statistically significant.

## Results

### Basic characteristics

Comparison of the sarcopenic, sarcopenic obesity, and healthy participants concerning basic characteristics were presented in Table 1. Similar to our previous study, there is a significant difference between sarcopenic and healthy participants in the case of age (*P* < 0.0001), waist circumference (*P* < 0.0001), calf circumference (*P* < 0.0001), BMI (*P* < 0.0001), appendicular lean mass (*P* < 0.0001), fat free mass (*P* < 0.0001), fat mass (*P* < 0.0001), SMI (*P* < 0.0001), HGS (*P* < 0.0001), GS (*P* = 0.046), albumin (*P* = 0.005). In addition, we observed a significant difference between mean value of waist circumference (*P* < 0.0001), calf circumference (*P* < 0.0001), BMI (*P* < 0.0001), appendicular lean mass (*P* < 0.0001), fat free mass (*P* < 0.0001), fat mass (*P* < 0.0001), fat mass percentile (*P < 0.0001*), SMI (*P* = 0.002), HGS (*P* < 0.0001), fat mass/free fat mass ratio (*P < 0.0001*), creatinine (*P* = 0.008), and HDL (*P* = 0.002) between sarcopenic obesity and healthy participants.

### Genotypes and allele frequency of Tp53 rs1625895

Genotypes and alleles frequency of TP53 rs1625895 were measured using chi-square test and is shown in Table 2. The genotype distribution in sarcopenic group (sarcopenic/ healthy individuals) was GG = 41.6%, AG = 50%, and AA = 8.4%. Also, the minor allele frequency in sarcopenic group was 33.3%. No significant variances in regard to genotypes and alleles detected between sarcopenic and healthy individuals. In addition, the distribution of TP53 rs1625895 in sarcopenic obesity group (sarcopenic obesity/ healthy) was GG = 41.3%, AG = 49.5%, and AA = 9.2%. The minor allele frequency in sarcopenic obesity group was 34%. A significant difference between sarcopenic obesity and healthy individuals in the case of alleles frequency was detected. G allele frequency was considerably greater in sarcopenic obesity group [*P* = 0.037, OR (CI 95%) = 1.9 (1.03–3.5)]. The genotype frequency in controls of both sarcopenic (*P* = 0.055) and sarcopenic obesity (*P* = 0.066) groups were in consistent with Hardy–Weinberg equilibrium.

**Table 2:** Genotypes and allele frequency of TP53 rs1625895 in the studied population. *P*-values< 0.05 were shown in bold

Genotypes	Sarcopenic (*n* = 45)	Healthy (*n* = 131)	*P* -value	OR (95% CI)	SO (*n* = 33)	Healthy (*n* = 131)	*P*- value	OR (95% CI)
AA	5 (11.1%)	10 (7.6%)	Reference		2 (6.1%)	10 (7.6%)	Reference	
AG	21 (46.7%)	73 (55.7%)	0.36	0.57 (0.18–1.9)	11 (33.3%)	73 (55.7%)	0.74	0.75 (0.14–3.9)
GG	19 (42.2%)	48 (36.7%)	0.7	0.8 (0.2–2.6)	20 (60.6%)	48 (36.7%)	0.37	2 (0.40–10.37)
Alleles								
A	31 (34.4%)	93 (35.4%)	Reference		15 (22.7%)	93 (35.4%)	Reference	
G	59 (65.6%)	169 (64.6%)	0.86	1 (0.6–1.7)	51 (77.3%)	169 (64.6%)	**0.048**	1.9 (1–3.5)
HWE	0.055				0.066			

Abbreviations: *SO* sarcopenic obesity; *WC* waist circumference; *CC* calf circumference; *BMI* body mass index; *FFM* fat-free mass; *FM* fat mass; *ALM* appendicular lean muscle mass; *SMI* skeletal muscle mass; *HGS* hand grip strength; *GS* gait speed; *HWE* Hardy–Weinberg equilibrium. Chi square test was used. *P*-value< 0.05 are considered statistically significant (shown in bold)

### The effect of Tp53 rs1625895 polymorphisms on anthropometric and biochemical parameters

Linear regression analysis was executed to determine the association between TP53 rs1625895 polymorphisms (GG vs AA/AG and AG/GG vs AA) and anthropometric and biochemical parameters in our studied population (Table 3). BMI (*P* = 0.049) and LDL (P = 0.04) were significantly different between genotypes when GG was compared to AA/AG genotype. We observed that the mean of BMI in individuals with GG genotype was higher than AG and AA genotypes. No significant differences were found in the case of other anthropometric and biochemical parameters in both genotype comparison (GG vs AA/AG, and AG/GG vs AA).

**Table 3:** Linear regression analysis for association between TP53 rs1625895 polymorphisms, and anthropometrics and biochemical parameters in our studied population. *P* values were adjusted for age

Data	Genotypes					
Variables	*AA vs AG/GG			#GG vs AA/AG		
	Beta	SE	*P*-value	Beta	SE	*P*-value
WC, cm	−1.354	1.270	0.288	−1.234	0.627	0.174
CC, cm	−0.053	0.452	0.907	−1.767	1.294	0.236
BMI, kg/m^2^	−0.839	0.618	0.176	−0.545	0.459	**0.049**
ALM, kg	−0.262	0.672	0.697	0.329	0.685	0.631
FFM, kg	−0.090	1.169	0.938	0.014	1.193	0.991
FM, kg	−1.191	1.147	0.300	− 1.525	1.168	0.193
FM, %	−1.165	1.284	0.365	1.498	1.308	0.254
SMI, kg/m^2^	−0.100	0.158	0.526	0.015	0.162	0.927
HGS, kg	0.911	2.532	0.719	2.906	2.576	0.261
GS, m/s	0.114	0.086	0.188	0.111	0.088	0.208
FM/FFM	−0.034	0.032	0.289	−0.042	0.033	0.194
Albumin, g/dl	0.020	0.033	0.541	−0.010	0.033	0.769
Creatinine, mg/dl	0.035	0.035	0.312	0.025	0.035	0.478
BUN, mg/dl	0.210	0.776	0.786	0.168	0.792	0.832
FBS, mg/dl	0.604	5.254	0.909	−2.635	5.359	0.632
Cholesterol, mg/dl	8.395	5.972	0.161	9.453	6.088	0.122
LDL-C, g/dl	7.644	4.964	0.125	10.398	5.042	**0.04**
HDL-C, mg/dl	0.352	1.878	0.851	−0.575	1.916	0.765
Triglycerides, mg/dl	13.877	11.052	0.211	8.900	11.306	0.432

Abbreviations: *SE* Standard Error; *WC* waist circumference; *CC* calf circumference; *BMI* body mass index; *FFM* fat-free mass; *FM* fat mass; *ALM* appendicular lean muscle mass; *SMI* skeletal muscle mass; *HGS* hand grip strength; *GS* gait speed. *P*-value<.0.05 are considered statistically significant (shown in bold)

### The association of Tp53 rs1625895 genotypes with the risk of sarcopenic obesity

Logistic regression analysis in unadjusted (model 1) and adjusted for age (model 2) models was done to find which genotype of TP53 rs1625895 may increase the risk of sarcopenic obesity (Table 4). The results revealed when GG genotype was compared to AA/AG genotype, GG increased the risk of sarcopenic obesity in unadjusted model [*P* value = 0.016, OR (CI 95%); 2.5 (1.19–5.48)], and in adjusted model [*P* value = 0.011, OR (CI 95%); 2.72 (1.25–5.91)]. Similar results were perceived when GG/AG genotype was compared to AA genotype. GG/AG genotype increased the risk of sarcopenic obesity in unadjusted model [*P* value = 0.035, OR (CI 95%); 2.32 (1.06–5.09)], and in adjusted model [*P* value = 0.028, OR (CI 95%); 2.43 (1.10–5.36)].

**Table 4:** Logistic regression analysis for association of TP53 rs1625895 genotypes with the risk of sarcopenic obesity in unadjusted mode (model 1) and adjusted for age model (model 2)

Data	Sarcopenic obesity			
	Model 1		Model 2	
TP53 rs1625895	OR (CI 95%)	P-value	OR (CI 95%)	*P*-value
*AA+AG vs GG	2.5 (1.19–5.48)	**0.016**	2.72 (1.25–5.91)	**0.011**
#AA vs AG + GG	2.32 (1.06–5.09)	**0.035**	2.43 (1.10–5.36)	**0.028**

* AA+AG was set as reference genotype and compared to GG genotype

# AA genotype was set as reference and compared to AG + GG genotype

*P*-value<.0.05 are considered statistically significant (shown in bold)

## Discussion

This is a novel study that investigated whether TP53 rs1625895 (IVS6 + 62A > G) is associated with the increased risk of sarcopenia and sarcopenic obesity. The results of this study confirmed the outcomes in our previous study. Similarly, we displayed that age, waist circumference, calf circumference, BMI, ALM, FFM, FM, FM %, SMI, HGS, GS, FM/FFM, Albumin, Creatinine was significantly differed between sarcopenic and healthy individuals. We also observed a significant difference between sarcopenic obesity and healthy in the terms of waist circumference, calf circumference, BMI, ALM, FFM, FM, FM %, SMI, HGS, GS, FM/FFM, Creatinine and HDL. The major findings of our study is that G allele is the risk factor of sarcopenic obesity. In addition, carriers of G allele (GG + AG) had higher risk of sarcopenic obesity compared to non-carriers (AA). We also observed a significant association between TP53 rs1625895 polymorphism and BMI and LDL in our studied population. However, no significant association between TP53 rs1625895 polymorphism and the risk of sarcopenia was detected.

In the last decades, some studies investigated the role of various genes and signaling pathways that was susceptible in progression of obesity, osteoporosis, and insulin resistance [26]. However, the genetic background of sarcopenia and sarcopenia related obesity in undefined yet.

The TP53 is a human gene that has been studied highly and comprehensively studied human gene since 40 years ago [27]. TP53 plays a critical role in inhibiting cancer progress, and is considered as the “guardian of the genome” [28]. Previous study recommended that expression and activation of p53 protein play a role in maintaining muscle homeostasis like myoblasts differentiation [15]. It was also demonstrated that although p53-defective myoblasts had normal cell cycle but differentiation of these cells into myocytes and myotubes was deceased so that, it could be a possible mechanism underlying the role of p53 in skeletal muscle differentiation [29].

Now, several TP53 polymorphisms like intronic variations have been defined and considered as important regulator of TP53 expression that can stimulate the synthesis of TP53 protein with altered structural and functional characteristics. It is supposed that intronic variations may modify the gene function by interfering with RNA splicing and via the interaction between the DNA strand and protein molecules [30]. On of this functional polymorphism is located in the intron 6 of the TP53 gene (rs1625895) and lead to the change of guanine for adenine on the site of the restriction endonuclease *MspI*. This polymorphism was established to modify the expression of protein p53 [31], however, the exact mechanism is unclear. It was proposed that rs1625895 may influence splicing or transcription factor binding or may be a target site for regulatory miRNAs [30].

The association between TP53 rs1625895 polymorphism (IVS6 + 62A > G) and susceptibility to sarcopenia and sarcopenia-related obesity has not been investigated previously. Here, we detected that G allele increased the risk of sarcopenic obesity of about 1.9 fold higher than A allele. Furthermore, in logistic regression analysis, we observed that when GG genotype was compared to AG/AA genotype, the risk of sarcopenic obesity increased 2.5 and 2.7 fold in unadjusted and adjusted models, respectively, and when GG/AG genotype was compared to AA genotype, the risk of sarcopenic obesity increased about 2.3 and 2.4 fold in unadjusted and adjusted models, respectively. This results revealed that the carriers of G allele independent of age increased the sarcopenic obesity while no significant influence of this polymorphism on the risk of sarcopenia was detected.

Strong evidence demonstrated the fundamental importance of p53 in metabolic diseases for example cardiovascular disease, obesity, and type 2 diabetes [32]. p53 is known as a negative regulator of adipogenesis in vitro, and also it was reported that p53 levels in white adipose tissue are augmented in diet-induced and genetic obesity mouse models and in obese humans [33]. Elevated expression and activity of p53 was proven to occurs in the adipose tissue of obese mice [34]. In 2019, Sabir et al. observed that p53 rs1042522 mutant allele is more frequent among obese individuals in Saudi population and suggested that p53 may be considered as genetic modifier for obesity development [35]. The p53 rs1042522 polymorphism (Pro72Arg) impression on body metabolism was also inspected in humanized p53 mice [36]. It was revealed that under high fat diet, mice carrying the p53 rs1042522 mutant allele exhibited higher weight gain, associated with higher fat mass, adipose tissue immune cell infiltration, hepatic steatosis and fibrosis, and insulin resistance [33]. In another study, Shafiee et al. 2018 reported that P53 besides Neurotrophic Receptor Tyrosine Kinase 1 (NTRK1) and Cullin 3 (CUL3) in the protein-protein interaction (PPI) network involved in lipid storage in older women [15]. Wang et al. 2009 found that PPAR pathway activate P53 through accretion of PLINs and additional fatty acids as toxic lipid intermediates, and decline the muscle volume in older people [37].

Wu et al. (2002) in a functional in vitro study suggested that mutant variant in the TP53 rs1625895 significantly reduced the ability of TP53 to regulate DNA repair processes [25]. One explanation for the link between TP53 rs1625895 and the increased risk of sarcopenia related obesity in our study is that mutation in TP53 rs1625895 like p53 rs1042522 results in impaired function of TP53 as a negative regulator of adipogenesis, however the exact mechanism is unclear. Since it was reported that elevated expression and activity of p53 is well known to occur in the adipocytes [38], decreased expression and activity of P53 as a result of functional mutation may lead to increased rate of adipogenesis. In addition, aging beside calorie overload, cellular senescence, or cardiac dysfunction is believed as metabolic stressors which could induce p53 expression in visceral WAT [33]. So that, it may be concluded that individuals with mutation in the p53 gene have defects in protection against aging-induce adipogenesis.

In addition, we found the individuals carrying TP53 rs1625895 mutant allele had higher BMI compared to non-carriers. Di Renzo et al. 2014 reported that according to BMI, 4.54% of individuals in studied population were obese and sarcopenic. They also suggested that up to 20% of sarcopenia frequency might be declined by appropriate approaches for managing obesity. Similar to our results, a genome-wide association study indicated a strong association of the TP53 mutant variant (rs1042522 R72) with higher BMI [39]. Furthermore, Gloria-Bottini et al. described that *p53* variant Arg72 is accompanying with elevated risk of BMI and diabetes [40]. Also, a latest cohort study of over 2500 Dutch and Finnish individuals informed a significant association between R72 and higher waist circumference [12].

In the current study, we also displayed a significant difference in the case of LDL level between genotype groups. Subjects with GG genotype had lower LDL level compared to AG/AA genotype. According to our best knowledge, there is no study on association betweenTP53 rs1625895 and lipid levels. Sabir et al. 2019 reported that Arg72 variant of TP53 increased BMI, W/H ratio, cholesterol, LDL level, and insulin levels [35]. Although mutations in p53 could interrupt the fatty acid oxidation function [41, 42], the exact mechanism between LDL level and TP53 rs1625895 is unclear.

As a limitation of our study we can point to the relatively small sample size that may affect the statistical power of associations of TP53 rs1625895 polymorphism with the risk of sarcopenia and sarcopenic obesity. Persons enrolled in the current study were nominated from a cohort study from the South of Iran and may not be considered as the general population of Iranian old adults. As a strength, this is a former study on association of TP53 rs1625895 polymorphism with risk of sarcopenia and sarcopenic obesity. Since, Race and ethnicity may clarify some of the high variation of occurrence frequency for sarcopenia and sarcopenic obesity, and it is confirmed that body composition differs between major races [38], finding the genetic factor that influence the susceptibility to these two traits in different populations is crucial. So that, as another strength, in this study for the first time the association of a polymorphism with the risk of sarcopenia and sarcopenic obesity in Iranian population was investigated.

## Conclusions

In summary, for the first time we reported the association between TP53 rs1625895 polymorphism and the risk of sarcopenic obesity. We identified that G allele could possibly increase the risk of sarcopenic obesity. We also demonstrated that individuals carrying G allele increased the risk of sarcopenic obesity compared to non-carriers. Moreover, the significant impact of TP53 rs1625895 polymorphism on BMI and LDL was recognized. No significant relation between TP53 rs1625895 polymorphism and susceptibility to sarcopenia was detected.

## Data Availability

The datasets generated during and analyzed during the current study are not publicly available due to [according to protect the patient information] but are available from the corresponding author on reasonable request.
