# Correction: The association between TP53 rs1625895 polymorphism and the risk of sarcopenic obesity in Iranian older adults: a case-control study

**DOI:** 10.1186/s12891-022-05682-2

**Published:** 2022-08-18

**Authors:** Nima Montazeri-Najafabady, Mohammad Hossein Dabbaghmanesh, Nasrin Nasimi, Zahra Sohrabi, Nazanin Chatrabnous

**Affiliations:** 1 Endocrinology and Metabolism Research Center, Shiraz University of Medical Sciences, Shiraz, Iran; 2 Nutrition Research Center, Shiraz University of Medical Sciences, Shiraz, Iran

**Correction: BMC Musculoskelet Disord 22, 438 (2021)**

**https://doi.org/10.1186/s12891-021-04314-5**

Following the publication of the original article [1] the authors would like to correct a typing error of the first author’s last name to “Montazeri-Najafabady” instead of “Montazeri-Najababady”.

The original article [1] has been updated.
